# A new emergency in oncology: Bone metastases in breast cancer patients (Review)

**DOI:** 10.3892/ol.2013.1372

**Published:** 2013-06-04

**Authors:** TONI IBRAHIM, LAURA MERCATALI, DINO AMADORI

**Affiliations:** Osteoncology and Rare Tumors Center, IRCCS Istituto Scientifico Romagnolo per lo Studio e la Cura dei Tumori (IRST), Meldola I-47014, Italy

**Keywords:** breast cancer, bone metastases, skeletal-related events, multidisciplinary approach

## Abstract

Breast cancer (BC) is the most common tumour in females and as a result, the management of such patients is a major public health issue. A high percentage of BC patients develop bone metastases (BMs), occasionally even several years following the initial diagnosis. BMs are responsible for high morbidity and a reduced quality of life with the onset of various clinical complications defined as skeletal-related events (SREs), including pathological fractures, spinal cord compression, hypercalcaemia, bone marrow infiltration and severe bone pain, requiring palliative radiotherapy. Such complications reduce functional independence and quality of life, decrease survival rates and increase healthcare costs. The current treatment for metastatic BC aims to achieve meaningful clinical responses, an improved quality of life, long-term remission, prolonged survival and in a small percentage of cases, a complete cure. The treatment of this malignancy has become progressively complex, including well-known antitumour agents or bone-targeted molecules aimed at preventing bone complications and improving patient quality of life and the treatment outcome of a multidisciplinary programme. The importance of a multi disciplinary approach in the management of BMs is also widely accepted. The major complication of BMs are SREs which are responsible for reducing prognoses and patient quality of life and are correlated with high rates of hospitalisation with the subsequent social and economic consequences. For these reasons, it is crucial to prevent where possible or to identify and treat SREs promptly in an attempt to mitigate the ever-increasing clinical and economic burden.

## Contents

1. Natural history of bone metastases in breast cancer
2. Complications of bone metastases
3. Classification of patients
4. Treatment of bone metastases

## 1. Natural history of bone metastases in breast cancer

Breast cancer (BC) is the most common tumour to affect the female population in the Western world with a worldwide prevalence of 1.5 million throughout industrialised countries. BC mortality rate is second only to lung cancer in the United States and Europe and its high incidence and prevalence makes it a major public health problem. Each year BC accounts for the diagnosis and mortality of >1,000,000 and >400,000 females worldwide, respectively. BC occurrence in males is markedly lower, accounting for ∼1% of all BC patients. Overall, the incidence of BC increases with age and particularly rapidly during the fourth decade of life and continues to increase thereafter, albeit more slowly in the fifth, sixth and seventh decades. In the United States, ∼75% of new BC diagnoses occur in females >50 years old and the lifetime risk of a BC diagnosis is ∼12.5%. The incidence rates for BC are similar in North America and the majority of other Western industrialised nations. In Japan and other Far Eastern countries, however, absolute incidence rates are lower for each age group and overall, Japanese females are five times less likely to develop BC than American females (1).

Metastases from cancer are the most common form of malignancy involving the bone with the highest prevalence in breast, prostate and lung cancers. Advances made in the diagnosis and treatment of tumours, including surgery, chemotherapy, biotherapy and radiotherapy, have increased survival rates in cancer patients over the last 20 years. Following the lung and liver, bone is the third most common site of metastasis and bone metastases (BMs) are responsible for high morbidity in cancer patients due to their epidemiological and clinical impact (2). The prognosis for BC depends on the stage at diagnosis, with the 5-year survival rates ranging between 84 and 18% in females diagnosed with stage I and IV of the disease, respectively. Despite the increasing incidence rate of BC, morbidity and mortality rates are beginning to decrease with substantial reductions in the middle age (∼25%) and to a lesser extent in old age (1).

At the diagnosis of BC, ∼5–6% of females present with distant spread, with bone being the most common site of metastatic lesion. Bone is the first site of metastasis in >50% of patients who relapse systemically and it is estimated that between 65 and 75% of BC patients who have a relapsed disease develop bone metastasis. Although any bone may be affected, the axial skeleton, including the skull, spine, ribs and pelvis, are the most frequent sites of involvement as compared to the extremities (3–5). For these and additional reasons, the current review reported that BMs are the leading cause of morbidity in patients with BC and may be observed as a new emergency.

The process that leads cancer cells to escape from the primary tumour to distant organs, where the cancer cells manage to grow and form a metastasis, is the most lethal development of cancer progression (6,7). Numerous events must occur to achieve metastasis formation, including cancer cell acquisition of an invasive phenotype that permits cells to detach from the primary tumour, usually through epithelial mesenchymal transition. Cells then reach the bloodstream or the lymphatic circulation system and eventually die or reach a second organ where the cells stop and colonise. Cancer cells from a specific site have a particular tropism versus preferential secondary organs (6,8). With regard to BC, the majority of individuals with advanced disease develop liver, lung, brain and primarily BMs. To stop and grow in these areas, cancer cells must acquire multiple alterations and change their expression profile to achieve a bone-like phenotype, including acquiring specific properties of bone cells to allow them to survive in bone tissue. Previous technological progress in functional genomics and genetic manipulation of cancer cells in animal metastasis models have led to the identification and analysis of tissue-specific metastasis genes (9). These genes may be significant for mediating tumour-stroma interaction during metastasis and may be observed as a target for therapeutic interventions (10,11).

There are a number of retrospective studies with regards to the molecules involved in bone tropism already expressed in primary tumours (12). For example, in a previous paper, positive expression of CXCR4 and RANK in patients with metastasis to bone was significantly different to those with no evidence of disease (13). Metastatic recurrence is most common in cancer patients a number of years following surgery for the primary tumour. Tumour cells disseminate from the primary tumour at an early stage of the cancer progression and are capable of remaining as dormant solitary cells or micrometastases in distant sites until certain genetic or epigenetic events convert them into active, overt and fast-growing macrometastasis (9). When the tumour cells arrive in the bone microenvironment, cancer cells disrupt the physiological balance between bone resorption and formation, thereby creating mixed lesions which are predominantly lytic in BC. This classification is based exclusively on their radiographic appearance (14–16).

## 2. Complications of BMs

In BC patients with bone lesions, ∼25% are asymptomatic and the diagnosis is only made when tests are carried out for additional reasons or during primary tumour staging. In the remaining 75%, BMs are responsible for various clinical complications defined as skeletal-related events (SREs), including pathological fractures, spinal cord compression, hypercalcaemia, bone marrow infiltration and severe bone pain, requiring palliative radiotherapy (Table I) (2). Such complications are often devastating for individuals and substantially reduce their functional independence and quality of life, decrease survival rates (17,18) and increase healthcare costs (19).

Using the Danish National Patient Registry, which covers all Danish hospitals, of the 35,912 BC patients registered between 1st January, 1999 and 31st December, 2007, 178 (0.5%) presented with BMs at the diagnosis of the primary BC and of these, 77 (43.2%) developed an SRE during follow-up (20). A total of 1,272/35,690 (3.6%) BC patients without BMs at diagnosis developed BMs during a median follow-up of 3.4 years. Among these individuals, 590 (46.4%) subsequently developed an SRE during a median follow-up of 0.7 years. Incidence rates of BMs were highest in the first year following the primary BC diagnosis and particularly among patients with advanced BC at diagnosis. Similarly, incidence rates of the initial SRE were highest in the first year following the initial diagnosis of a BM (20).

A Canadian study evaluated the pattern of metastatic disease in 180 triple-negative, including estrogen and progesterone receptor- and HER2/neu-negative, BC patients who were compared with additional subgroups of BC (N = 1,428). The risk of developing BMs within 10 years of the diagnosis was 7–9% for all subgroups (21). A number of clinical trials have evaluated the efficacy of bisphosphonates in reducing skeletal complications in patients with BC and BMs (22,23). These studies showed that the median time to the first SRE was 13.9 months among bisphosphonate-treated females and 7.0 months in the placebo group (P=0.001) (23). The SREs that occurred in the placebo group were radiation to bone, pathological fracture, hypercalcaemia, surgery on bone and spinal cord compression (23).

## 3. Classification of patients

The classification of patients was established on a number of factors that are significant for evaluating prognosis and identifying the most suitable therapeutic strategies. The median survival between the diagnosis of metastatic disease and mortality have been reported as follows: 22 months, overall; 26 months, in patients with BMs only; 21 months, in patients with bone and visceral metastases and 18 months, in patients with visceral metastases only (24).

A previous study (25) showed that following a median follow-up of 28 months, 59.2% of patients with BC developed new metastases and progression occurred notably in non-skeletal sites, with the exception of individuals with previous BMs, which progressed prevalently to bone. The 2-year probability for disease progression control and survival was 0.19 (95%, CI 0.15–0.24) and 0.64 (95% CI, 0.58–0.69), respectively. The 2-year probability of mortality according to the presence of non-skeletal metastases and the time of appearance (previous or concurrent to BMs) was: 0.74 (95% CI, 0.67–0.79), for bone metastatic patients exclusively; 0.38 (95% CI, 0.25–0.51), for previous non-skeletal metastases and 0.56 (95% CI, 0.46–0.66) for concomitant non-skeletal and BMs (P<0.0001) (25). The presence of osteosclerosis as the predominant form of metastasis is correlated with improved survival in BM BC patients.

An additional factor that significantly affects survival is the presence of solitary or multiple metastatic skeletal lesions. A previous study (26) has shown that solitary metastatic bone lesions account for 41% of initially diagnosed metastatic bone lesions. Individuals with a solitary metastatic bone lesion, regardless of the site, have an improved prognosis compared to patients with multiple metastatic bone lesions. The time between the initial diagnosis and the development of skeletal metastasis was higher in patients with a solitary metastatic bone lesion. In addition, a solitary metastatic bone lesion was often resolved during treatment and complete remission was not unusual (26).

These variances may be due to biological differences, for example, patients who develop solitary skeletal metastasis have favourable biological factors when compared to those who develop multiple skeletal metastases at initial osseous metastatic events. Cox proportional hazards regression showed that estrogen and progesterone receptor status, disease-free interval (bone metastasis-free interval), first metastasis organs and the type of bone lesions (solitary vs. multiple) were independent prognostic factors.

In a univariate analysis of progression-free survival (PFS) and overall survival (OS), patients with metastasis at presentation with a single bone metastasis or with asymptomatic bone disease exhibited a higher PFS interval when compared with that of patients with metastasis at recurrence with multiple metastases or with symptomatic bone disease. Patients with an ECOG performance status score of 0 or 1 with a single metastasis or with asymptomatic bone disease have a higher OS time when compared with that of patients with a performance status score of 2 or 3 with multiple sites of bone metastasis or with symptomatic bone disease (27).

## 4. Treatment of BMs

The current treatment for metastatic breast cancer (MBC) aims to achieve meaningful clinical responses, improved quality of life, long-term remission, prolonged survival and in a small percentage of cases, a complete cure. BMs are a major cause of morbidity in these individuals and the treatment of this malignancy has become progressively complex, including well-known antitumour agents or bone-targeted molecules aimed at preventing bone complications and improving patient quality of life and the treatment outcome of a multidisciplinary programme (Table II). The importance of a multidisciplinary approach in the management of BMs is also widely accepted. The main complications of BMs are SREs which are responsible for reducing prognoses and patient quality of life and are correlated with high rates of hospitalisation, with the ensuing social and economic consequences. For these reasons it is essential to prevent or to identify and treat SREs promptly in an attempt to mitigate the ever-increasing clinical and economic burden.

The prognostic factors reported in the present review addressed the classification, management and best treatment choices for individuals, particularly the choice of the specific antitumour agent and interdisciplinary programme.

Patients with pessimistic prognostic factors must be advised to receive treatments with few side effects, complications and of minimal invasiveness. However, for patients with positive prognostic factors, in addition to medical treatments, it is essential that other treatments are integrated to reduce the complications from BM and increase survival with a good quality of life.

The introduction of additional effective systemic therapies over the last two decades has achieved substantial improvements in clinical outcomes and the majority of patients now live with metastatic secondary BC for a number of years. Various antitumour agents are used in clinical practice and targeted biological therapies are of increasing importance for the management of various BC subtypes (28).

Hormonal therapy remains significant for the management of MBC luminal A and B. Studies on the management of ErbB2- and hormone receptor-positive MBC by a combination of hormone manipulation and targeted anti-ErbB2 therapy have previously received regulatory approval in Europe and the USA. In addition, aromatase inhibitors (AI) have been extensively studied in this setting and in postmenopausal females, AI are the first line of treatment for untreated patients or those who have received previous AI treatment and progressed following 12 months of adjuvant therapy. A higher disease-free interval and the absence of visceral disease are correlated with improved response. If tumours recur within <12 months it is advisable to initiate treatment with Tamoxifen (TAM) or the estrogen receptor antagonist Fulvestrant (FUL). In the second line setting the primary option, following progression, is the administration of FUL or TAM and in the third line setting the reintroduction of AI is considered an acceptable option. In premenopausal females who have not received prior treatment or who have progressed following 12 months of adjuvant treatment, it is advisable to initiate treatment with a combination of TAM and a luteinizing hormone-releasing hormone (LHRH) analog. If treatment fails with the use of this combination, megestrol acetate or an LHRH agonist in addition to an AI may be reasonable alternatives (29).

Chemotherapy, including anthracyclines, taxanes, vinorelbine, capecitabine, gemcitabine and platinum agents, is usually reserved for individuals with a disease that is unresponsive to endocrine agents, with rapidly progressive visceral disease or BM with pessimistic prognostic factors (28). Increasing controversy surrounds the use of newer agents, including nab-paclitaxel, ixabepilone, eribulin, PARP inhibitors, bevacizumab and other antiangiogenetic agents. However, improved understanding of pathophysiology has suggested the likely introduction of newer anti-ErbB2 agents, including lapatinib, pertuzumab T-DM1 and neratinib (30).

Bisphosphonates have significantly changed the natural history of BMs by reducing SREs and have made significant improvements in the quality of life and treatment outcome for patients with MBC. In addition, newer anti-RANK ligand antibodies show promising results and the significant advances that have been made in the understanding of the molecular biology of BC must lead to an improvement in the treatment of MBC through the identification of a number of addition bone-targeted molecules (31). In accordance with available studies, the ESO-MBC Task Force maintains its original recommendation statement: ‘A small but very important subset of MBC patients, for example, those with a solitary meta static lesion, can achieve complete remission and a long survival. A more aggressive and multidisciplinary approach must be considered for these selected patients. A clinical trial addressing this specific situation is needed.’ (32).

Metastatic bone pain is severe, progressive, multifocal and established on multiple pathogenetic mechanisms. For these reasons, its management must rely on systemic drug therapy, including NSAIDs, opioid and adjuvant drugs, supplemented, where necessary, with additional multidisciplinary forms of treatment, for example, palliative or curative surgery, local curative or palliative radiotherapy, arterial embolization, kyphoplasty/vertebroplasty or systemic radiometabolic treatment with radioisotopes, including Samarium-153 or Strontium or Rhenium-186. These treatments may be significant for pain control and the prevention of SRE when integrated with each other and medical anticancer therapies. Integrated rehabilitative therapy has an essential role in managing BMs and the use of aids, including corsets, collars and crutches, is aimed at ensuring functional recovery and avoiding SREs (33).

The management of BMs in BC requires a multidisciplinary approach involving a team of specialists involved in the diagnosis, treatment and assistance of patients. However, the management of this disease in a number of countries remains fragmented and unsatisfactory. Patients are frequently constrained to making referrals to a series of specialists, often with lengthy waiting lists, creating great psycho-physical stress and leading to poor healthcare continuity. The solution would be to establish dedicated multidisciplinary osteoncology centres with the aim of proposing a customised multidisciplinary approach that integrates clinical practice and study, improves the use of human and economic resources, increases the quality of services offered to individuals and reduces the risk of SREs (34). In conclusion, the present review has reported that BMs in BC is a new emergency which requires not only new antitumour agents and bone-targeted therapy but also an interdisciplinary approach involving oncologists, radiotherapists orthopedists, palliativists, physiatrists, radiologists, nuclear medicine specialists and others.

## Figures and Tables

**Table I.** Complications of bone metastases.

Pain
Pathological fractures
Spinal cord compression
Hypercalcemia
Bone marrow suppression

**Table II.** Treatment of bone metastases.

Medical treatment
Antitumor agents
Chemotherapy
Endocrine therapy
Biotherapy
Bone-targeted therapy
Bisphosphonates
RANK-L antibody
Palliative care
Analgesic drugs
Simultaneous supportive care
Radiotherapy
Orthopedic surgery
Interventional radiology
Radiometabolic treatment
Rehabilitation

RANK-L, receptor activator of nuclear factor-κB ligand.
